# Recurrent headache and visual symptoms in a young man: a rare neuronal intranuclear inclusion disease case report

**DOI:** 10.1186/s12883-022-02936-3

**Published:** 2022-11-02

**Authors:** Ning Su, He-Jiao Mao, Chen-Hui Mao, Li-Ying Cui, Yi-Cheng Zhu, Yan Zhou, Jun Ni

**Affiliations:** Department of Neurology, State Key Laboratory of Complex Severe and Rare Diseases, Peking Union Medical College Hospital, Chinese Academy of Medical Science and Peking Union Medical College, Beijing, 100730 China

**Keywords:** Neuronal intranuclear inclusion disease (NIID), Headache, Visual symptoms, Migraine with aura, Case report

## Abstract

**Background:**

Neuronal intranuclear inclusion disease (NIID) is a progressive neurodegenerative disease. Patients with NIID may present with heterogeneous clinical symptoms, including episodic encephalopathy, dementia, limb weakness, cerebellar ataxia, and autonomic dysfunction. Among the NIID cases reported in China, patients often have complicated and severe manifestations. Therefore, many clinicians do not consider the disease when the patient presents with relatively minor complaints.

**Case presentation:**

We present the case of a 39-year-old man showing migraine-aura-like symptoms for the past 3 years. Brain magnetic resonance imaging (MRI) revealed hyperintense signals in the splenium of the corpus callosum and corticomedullary junction on diffusion-weighted imaging (DWI) over time. In addition, brain atrophy that was not concomitant with the patient’s age was detected while retrospectively reviewing the patient’s imaging results. Genetic analysis and skin biopsy confirmed a diagnosis of NIID. The patient was treated with sibelium, and the symptoms did not recur.

**Discussion and Conclusions:**

Migraine-aura-like symptoms may be the predominant clinical presentation in young patients with NIID. Persistent high-intensity signals on DWI in the brain and early-onset brain atrophy might be clues for the diagnosis of NIID.

## Introduction

Neuronal intranuclear inclusion disease (NIID) is a chronic progressive neurodegenerative disease characterized by eosinophilic intranuclear inclusion bodies in the central and peripheral nervous systems and internal organs [1]. The clinical manifestations are highly heterogeneous, with dementia or cognitive decline being the most common, followed by episodic symptoms, including disturbance of consciousness, stroke-like episodes, and chronic headaches [2]. The pathology of NIID is characterized by the presence of eosinophilic intranuclear inclusion cells within the neurons and glia. Recently, genetic changes in the *NOTCH2NLC* genes have been found to cause NIID. Although there were records of Chinese NIID patients presenting with migraine, all of the patients showed complications with severe clinical manifestations such as epilepsy and disturbance of consciousness [3, 4]. Here, we report the case of an NIID patient with mild symptoms, predominantly migraine-aura-like symptoms.

## Case presentation

**Fig. 1:**
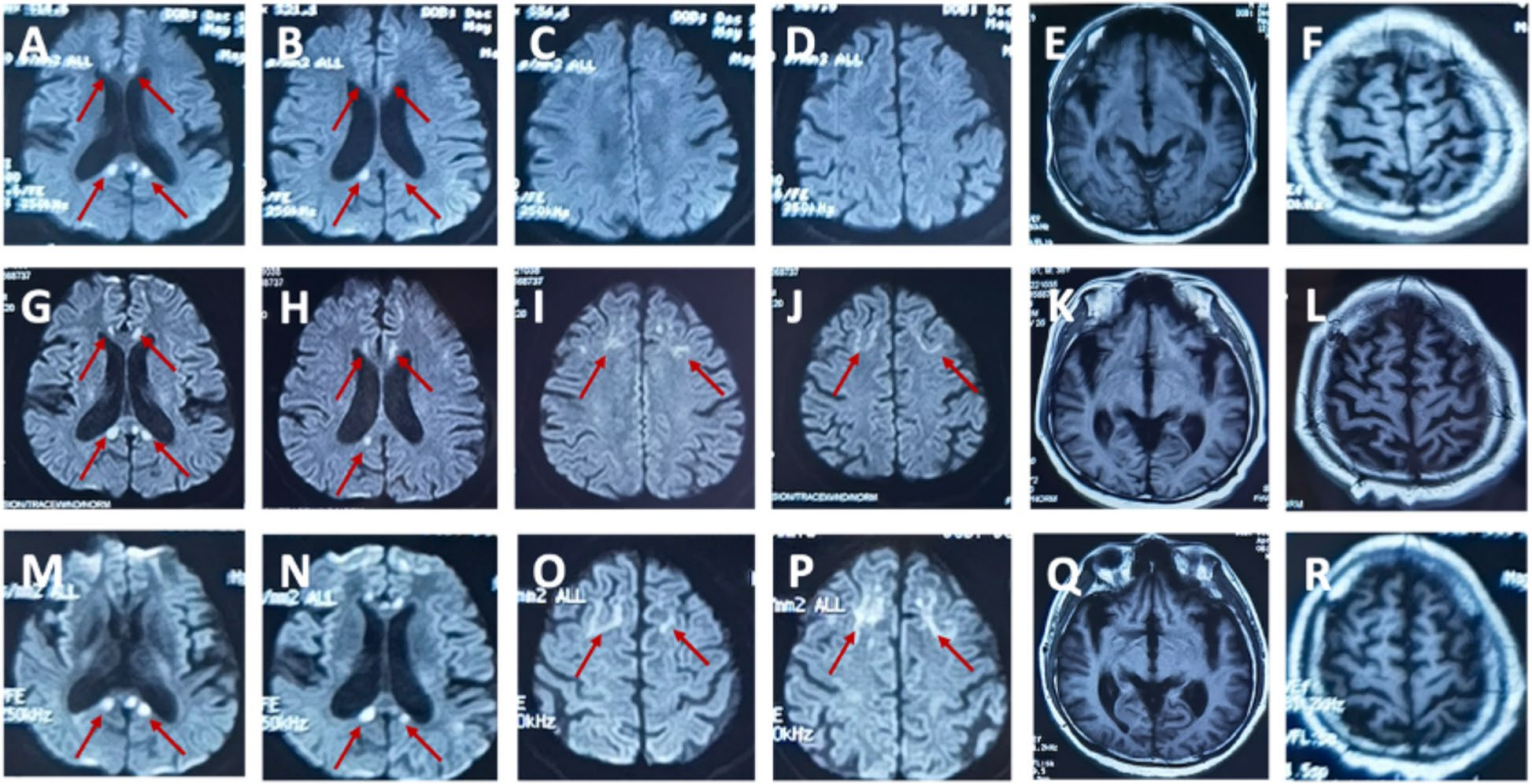
Brain MRI findings in the 39-year-old patient. Diffusion-weighted imaging (DWI) revealed hyperintense signals on the splenium of the corpus callosum and corticomedullary junction overtime. T1 showed brain atrophy in the bilateral temporal pole and cortex. (**A**-**F**) Imaging was performed on May 13, 2020. (**G**-**L**) Imaging was performed on September 23, 2020. (**M**-**R**) Imaging was performed on April 2, 2021

**Fig. 2:**
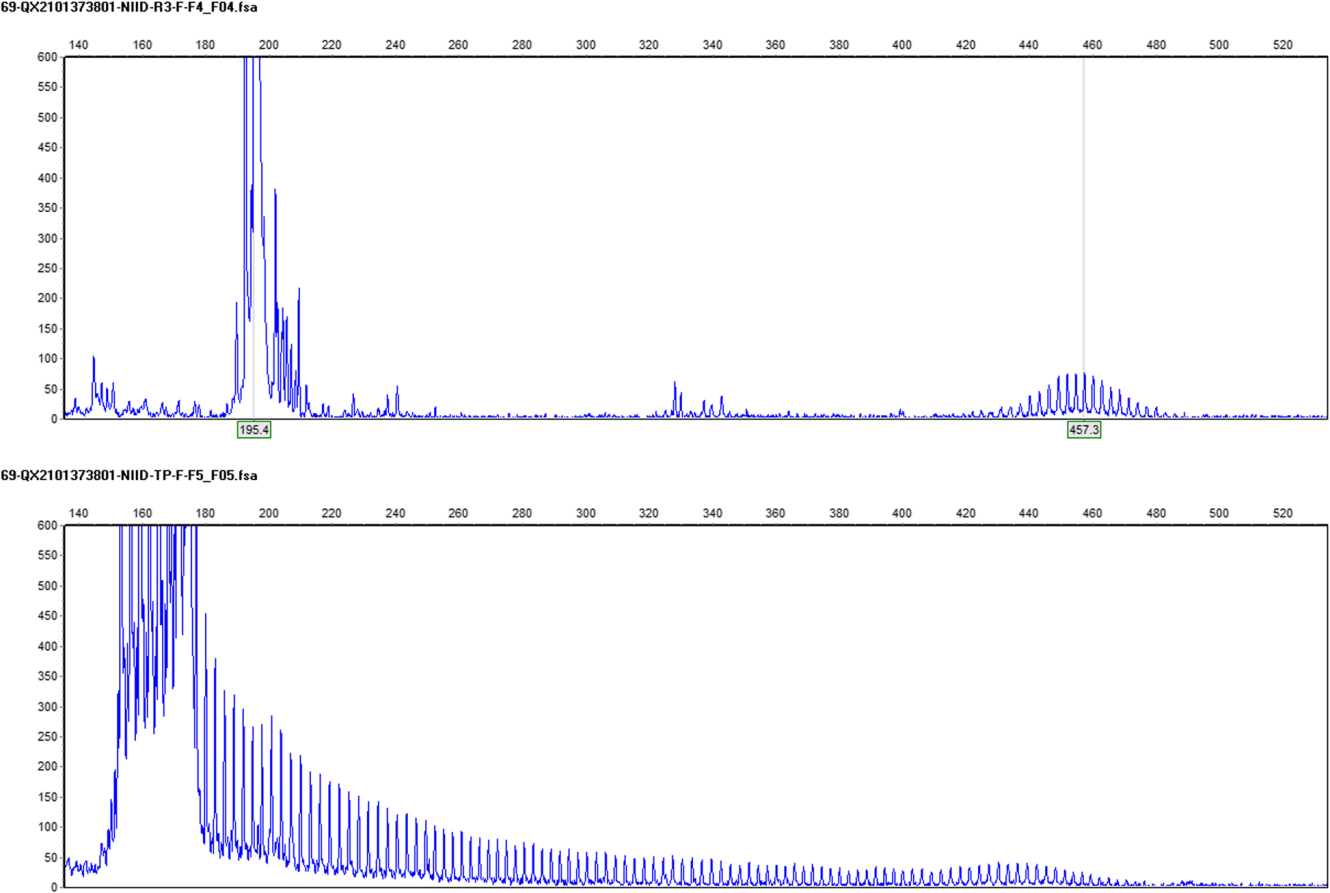
Genetic analysis results. Genetic analysis demonstrated an abnormal expansion of 101 GGC repeats in the *NOTCH2NLC* gene

**Fig. 3:**
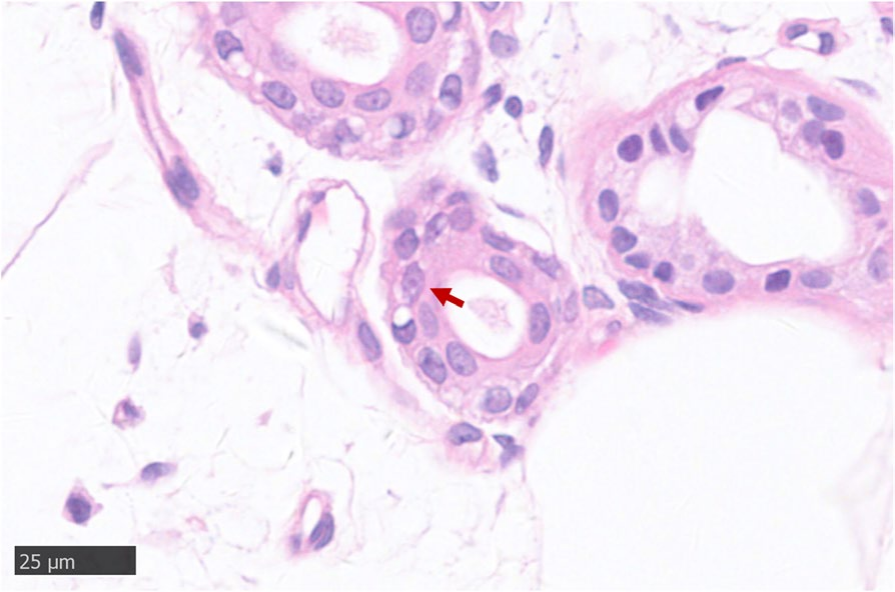
Hematoxylin–eosin staining of skin biopsy. It showed the morphology of neuronal eosinophilic hyaline intranuclear inclusions. The sections were observed under an upright clinical microscope (Nikon ECLIPSE Ci, Japan). Images were captured using a Nanozoomer slide scanner (Hamamatsu, Japan)

**Fig. 4:**
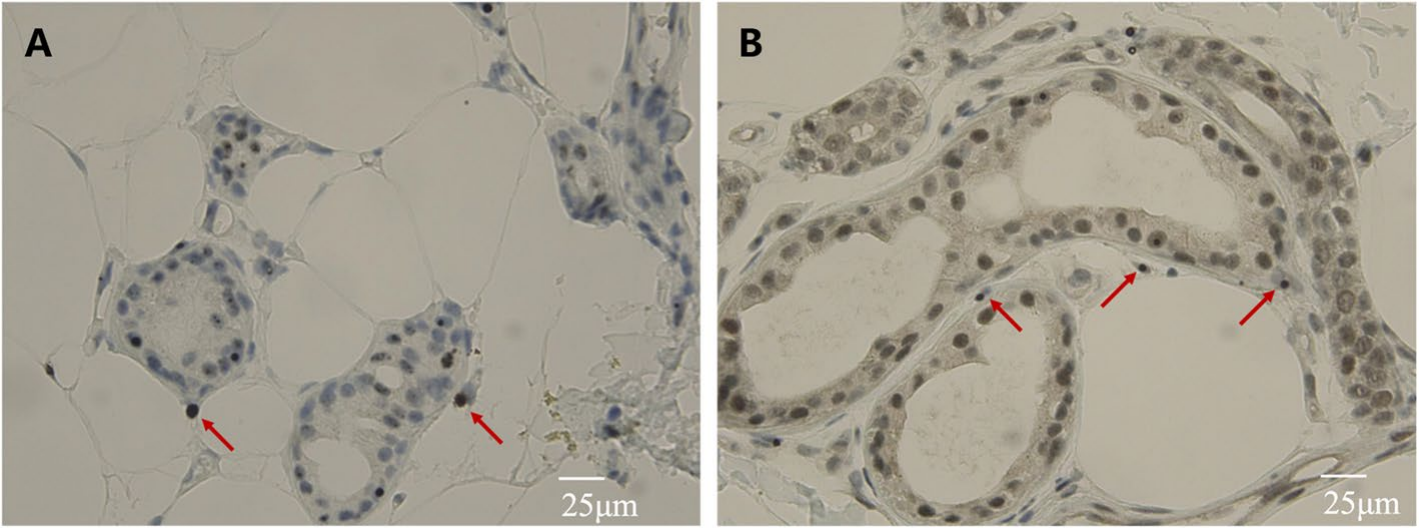
Immunohistology of skin biopsy. **A** Neuronal eosinophilic hyaline intranuclear inclusions were immunoreactive to P62. **B** Neuronal eosinophilic hyaline intranuclear inclusions were immunoreactive to ubiquitin. Images were captured using an upright clinical microscope (Nikon ECLIPSE Ci, Japan). Scale bar was edited using ImageJ

A 39-year-old man suffering from recurrent headache and paroxysmal visual symptoms for three years was admitted to our hospital. In 2018, the patient developed a persistent pulsatile and pinprick headache in the right temporal region. The visual analog scale (VAS) score [5] was 7/10. The headache was aggravated while coughing and was accompanied by photophobia. The patient claimed that the objects in the central area of the left visual field were blurred and deformed, followed by bilateral temporal visual field defects. The visual symptoms occurred minutes before the headache and sometimes in the postdromal phase 1–2 h after the headache commenced. The above symptoms lasted for 10 h and were relieved after taking a painkiller. The recurrence of headache with aura occurred once every two months. In 2019, the headache aggravated, with a VAS score of 10/10, and expanded to the right occipital region. Visual symptoms and headaches occurred simultaneously; the visual symptoms could be relieved after one night of sleep, and the headache could last for up to three days. These symptoms might have been triggered by staring under bright light. The patient’s medical history was unremarkable, and no history of migraine or family history of migraine was noted. Neurological examination showed no positive findings except for a score of 29 in the MMSE test and a score of 26 in the MoCA test. Initial brain magnetic resonance imaging (MRI) performed in 2020 revealed multiple focal high-intensity signals in the corpus callosum on diffusion-weighted imaging (DWI) (Fig. 1). Since the patient had recurrent headache and DWI lesions and CSF showed a mild inflammatory reaction, vasculitis was considered, and the patient was treated with antiplatelet and steroid medications in a local hospital; however, the treatment did not prevent the attacks. After admission, the patient underwent further examinations, including routine blood tests, liver and kidney function, and coagulation tests. All indicators were found to be normal. Autoantibody spectrum tests, including antiphospholipid antibody, antinuclear antibody, and systemic vasculitis antibody, showed negative results. Fundus examination did not reveal any abnormalities. Results of carotid artery ultrasound, transcranial Doppler (TCD) ultrasound, and brain magnetic resonance angiography (MRA) examinations were all normal. Brain MRI showed that the lesions in the corpus callosum remained the same as in the previous year (Fig. 1). Furthermore, hyperintense signals in the corticomedullary junction and mild brain atrophy were found (Fig. 1). The cerebrospinal fluid (CSF) profile was negative. Given the persistent DWI hyperintensity in the corticomedullary junction and corpus callosum, brain atrophy, and paroxysmal symptoms in this young patient, NIID was suspected. Genetic analysis revealed an abnormal expansion of 101 GGC repeats in the *NOTCH2NLC* gene (Fig. 2) [6, 7]. Genetic counseling was referred but was declined by patient and his family. Eosinophilic intranuclear inclusions in the nuclei of sweat gland epithelial cells and fibroblasts were detected through skin biopsy (Figs. 3 and 4), which confirmed the diagnosis of NIID. After the patient was discharged from the hospital, episodic symptoms appeared again after a cold, manifesting as a headache lasting for about 3 days after a visual aura. After oral administration of sibelium, the symptoms did not recur.

## Discussion and conclusions

The differential diagnosis for increased intensity of DWI signals in the corpus callosum consists of cerebral vascular disease (cerebral infarction), vasculitis (Susac syndrome), inflammatory or autoimmune disease (multiple sclerosis), and genetic metabolic disease (mitochondrial encephalomyopathy, leukodystrophy, Fragile X-associated tremor/ataxia syndrome) [1]. For this patient, the persistent hyperintensity in the DWI sequence lasting for 1 year did not correlate with the conversion of imaging after the acute infarction. Cervical and cerebral vascular screenings were normal, which also helped to exclude cervical or cranial vascular disorders. The patient had no clear evidence of retinal arterial occlusion or hearing loss, and inflammatory factors were negative; thus, Susac syndrome was unlikely [8]. There was no clue for space dissemination, the oligoband was negative in the CSF, and multiple sclerosis was not considered. Given that the patient also had hyperintensity on DWI in the corticomedullary junction and brain atrophy at a young age, hereditary degenerative disease was suspected. The definitive diagnosis relied on a skin biopsy, which showed infiltration of eosinophilic intranuclear inclusions in the nuclei of sweat gland epithelial cells (Figs. 3 and 4).

Symptomatic migraine has been systematically described in a recent publication. According to the SNNOOP10 list of red flags, the patient was suspected to have mild cognitive impairment based on MMSE and MoCA scores. Side-locked symptoms were not typical in this patient, as visual deficits occurred bilaterally, and the lesion was located on both sides of the brain [9, 10]. We could not exclude the causal relationship between primary NIID and headache or visual symptom episodes; however, the headache and visual symptoms deteriorated as the lesions spread in the brain. The temporal relationship between the symptoms and lesions provided indirect evidence.

NIID is considered a progressive neurodegenerative disease with infiltration of eosinophilic hyaline intranuclear inclusions in the central and peripheral nervous systems as well as visceral organs [1, 11–13]. Its pathogenesis is mainly related to genetic factors. The mechanism is still unclear, which may be related to the abnormal accumulation of proteins and the dysfunction of the intranuclear ubiquitin–proteasome pathway [14, 15]. NIID patients present with heterogeneous clinical symptoms including dementia, limb weakness, cerebellar ataxia, dystonia, parkinsonism, seizures, episodic encephalopathy, and autonomic dysfunction [2, 4]. Of the various clinical manifestations, migraine has been reported as a symptom. Wang et al. reported the first case of NIID in a 20-year-old man with hemiplegic migraine and seizure with abnormal expansion of 81 GGC repeats in the 5’UTR of the *NOTCH2NLC* gene [3]. Zhao et al. presented the case of an 86-year-old man with NIID and vestibular migraine-like attacks for more than 30 years, followed by essential tremor and dementia, and genetic testing revealed mutations in the *SCN5A* gene [4].

DWI showed high-intensity signals in the subcortical white matter area of the bilateral frontal lobes. A pathological study showed that the high-intensity signals on DWI and ADC at the corticomedullary junction corresponded with spongy changes in the pathology; the loss of myelin sheath and axon was also observed [16]. Since the intranuclear inclusion bodies were mainly deposited in astrocytes, it can be speculated that an astrocyte function disorder led to interstitial edema, resulting in high-intensity signals on DWI and ADC. Other diseases such as Creutzfeldt-Jakob disease, which is also accompanied by spongiosis, lack the pathological change leading to edema and show ADC hypointense, which can be distinguished from NIID.

The mechanisms of paroxysmal symptoms in NIID remain unclear. Of note, the vascular-related theory was a plausible explanation of the symptoms. One speculation is that cerebral hypoperfusion and vascular dysfunction contribute to the accumulation of eosinophilic inclusions, resulting in ischemic and hypoxic changes in neurons, finally leading to migraine attacks and NIID progression. Another hypothesis is that the accumulation of eosinophilic inclusions causes the development of vascular dysfunction, resulting in hypoperfusion and hypometabolism, which finally lead to migraine attacks [3]. When retrospectively reviewing the patient’s imaging findings, we found that brain atrophy, which was not concomitant with the patient’s age, was consistently present. This important finding gave us a clue to consider hereditary metabolic or degenerative diseases; thus, genetic screening was performed. A hypothesis explains brain atrophy accompanied by NIID. Diffuse vasospasm without ischemia in migraine [17] and blood–brain-barrier disruption[18] may explain the phenomenon of brain atrophy [3].

Sibelium was used to relieve headache symptoms in the patient. Sibelium is a drug with flunarizine hydrochloride as its active ingredient. Flunarizine hydrochloride is a selective calcium antagonist that blocks excess calcium ion flux through transmembrane proteins into cells, prevents excessive intracellular calcium load of neurons during ischemia and hypoxia, inhibits cerebral vasospasm, and promotes cell membrane stabilization. Numerous controlled clinical studies have established the efficacy of flunarizine, which has long been used in the prophylactic management of migraines [19, 20].

In conclusion, we have reported the case of a patient with NIID who mainly presented with migraine-aura-like symptoms, which are rare in NIID. NIID should be considered as a differential diagnosis when a patient presents with migraine aura-like symptoms, persistent high-intensity signals within the corticomedullary junction or corpus callosum on DWI, and early-onset brain atrophy. Timely gene analysis and skin biopsy are helpful for a definitive diagnosis.

## Data Availability

The original contributions presented in this study are included in the article, and further inquiries can be directed to the corresponding authors.

## Abbreviations

NIID: Neuronal intranuclear inclusion disease
MRI: Magnetic resonance imaging
DWI: Diffusion-weighted imaging
MMSE: Mini–Mental State Examination
MoCA: Montreal Cognitive Assessment
VAS: Visual analogue scale
TCD: Transcranial Doppler
MRA: Magnetic Resonance Angiography
CSF: Cerebrospinal fluid
